# Pyridine-4-carbaldehyde 4-phenylsemicarbazone

**DOI:** 10.1107/S1600536811013134

**Published:** 2011-04-16

**Authors:** Rafael Mendoza-Meroño, Laura Menéndez-Taboada, Eva Fernández-Zapico, Santiago García-Granda

**Affiliations:** a Departamento de Química Física y Analítica, Facultad de Química, Universidad de Oviedo - CINN, C/ Julián Clavería, 8, 33006 Oviedo, Spain

## Abstract

In the title compound, C_13_H_12_N_4_O, the semicarbazone fragment links a benzene and a pyridine ring in the structure. The crystal packing is stabilized by strong inter­molecular N—H⋯O hydrogen bonds, which connect two mol­ecules to form a *synthon* unit, and by N—H⋯N hydrogen bonds and weak C—H⋯π inter­actions. The mol­ecular conformation is stabil­ized by intra­molecular N—H⋯N and C—H⋯O inter­actions.

## Related literature

For related compounds and their biological activity, see: Pavan *et al.* (2010 ▶); Yogeeswari *et al.* (2005 ▶).
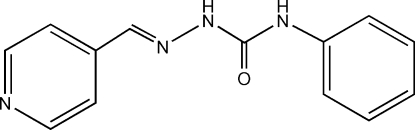


## Experimental

### 

#### Crystal data

C_13_H_12_N_4_O
*M* *_r_* = 240.27
Monoclinic, 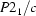

*a* = 9.2794 (6) Å
*b* = 10.3384 (8) Å
*c* = 12.8244 (8) Å
β = 100.744 (6)°
*V* = 1208.73 (15) Å^3^
*Z* = 4
Cu *K*α radiation
μ = 0.72 mm^−1^
*T* = 295 K
0.30 × 0.11 × 0.06 mm

#### Data collection

Oxford Diffraction Xcalibur Gemini R diffractometer
Absorption correction: multi-scan (*ABSPACK* in *CrysAlis PRO*; Oxford Diffraction, 2010 ▶) *T*_min_ = 0.888, *T*_max_ = 1.000
7257 measured reflections
2310 independent reflections
1702 reflections with *I* > 2σ(*I*)
*R*_int_ = 0.066

#### Refinement

*R*[*F* ^2^ > 2σ(*F* ^2^)] = 0.052
*wR*(*F* ^2^) = 0.143
*S* = 1.06
2310 reflections
176 parameters
H atoms treated by a mixture of independent and constrained refinement
Δρ_max_ = 0.20 e Å^−3^
Δρ_min_ = −0.17 e Å^−3^

### 

Data collection: *CrysAlis CCD* (Oxford Diffraction, 2010 ▶); cell refinement: *CrysAlis RED* (Oxford Diffraction, 2010 ▶); data reduction: *CrysAlis RED*; program(s) used to solve structure: *SIR92* (Altomare *et al.*, 1994 ▶); program(s) used to refine structure: *SHELXL97* (Sheldrick, 2008 ▶); molecular graphics: *ORTEP-3 for Windows* (Farrugia, 1997 ▶) and *Mercury* (Macrae *et al.*, 2008 ▶); software used to prepare material for publication: *WinGX* publication routines (Farrugia, 1999 ▶), *PLATON* (Spek, 2009) ▶ and *PARST95* (Nardelli, 1995 ▶).

## Supplementary Material

Crystal structure: contains datablocks global, I. DOI: 10.1107/S1600536811013134/fy2007sup1.cif

Structure factors: contains datablocks I. DOI: 10.1107/S1600536811013134/fy2007Isup2.hkl

Additional supplementary materials:  crystallographic information; 3D view; checkCIF report

## Figures and Tables

**Table 1:** Hydrogen-bond geometry (Å, °) *Cg*2 is the centroid of the C8–C13 ring.

*D*—H⋯*A*	*D*—H	H⋯*A*	*D*⋯*A*	*D*—H⋯*A*
N3—H3*N*⋯O1^i^	0.93 (2)	1.91 (2)	2.833 (2)	172 (2)
N4—H4*N*⋯N1^ii^	0.91 (2)	2.24 (2)	3.122 (3)	161.8 (19)
N4—H4*N*⋯N2	0.91 (2)	2.29 (2)	2.685 (2)	105.4 (16)
C13—H13⋯O1	0.93	2.31	2.854 (2)	117
C1—H1⋯*Cg*2^iii^	0.93	2.88	3.644 (2)	140

Symmetry codes: (i) 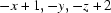; (ii) 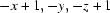; (iii) 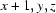.

**Fig. 1.**
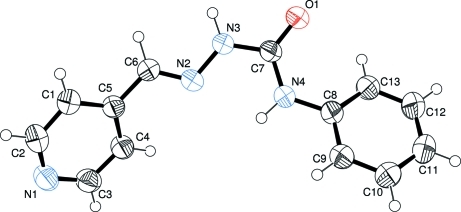
A view of the molecular structure of the title molecule showing the atom-numbering scheme. Displacement ellipsoids are drawn at the 50% probability level.

**Fig. 2.**
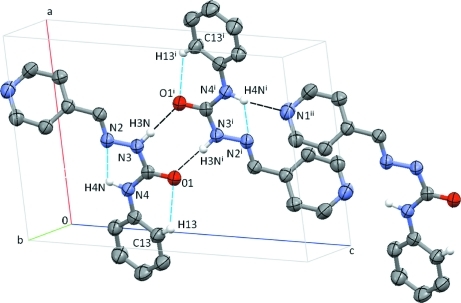
Part of the crystal packing showing the principal intermolecular and intramolecular hydrogen bonds. Displacement ellipsoids are drawn at the 50% probability level. H atoms not involved in hydrogen bonding have been omitted for clarity.

**Fig. 3.**
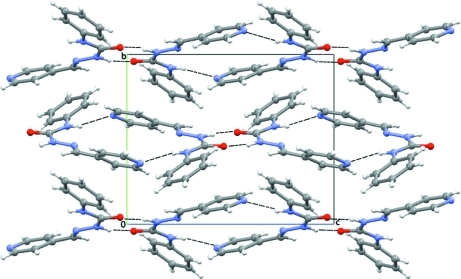
Packing diagram viewed along the c axis. Hydrogen bonds are indicated by dashed lines.

**Fig. 4.**
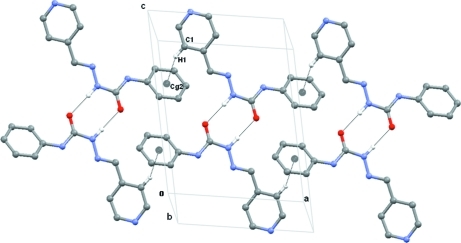
Intermolecular C—H···π interactions along ac plane. H atoms not involved in hydrogen bonding have been omitted for clarity.

## supplementary crystallographic information

### Comment

Semicarbazones and their metal complexes are important classes of compounds which have long attracted attention owing to their remarkable biological and pharmacological properties, such as antibacterial, antiviral, antineoplastic and anti *Mycobacterium tuberculosis* activity (Pavan *et al.*, 2010). Using the semicarbazone template (Yogeeswari *et al.*, 2005), significant anticonvulsant potential was demonstrated in epilepsy models for *aryl* semicarbazones. In view of the importance of these compounds, a new semicarbazone (I) has been synthesized, and its crystal structure is reported here (Fig. 1).

The configuration of (I) is E with respect to the C6═N2 bond. The pyridine and benzene rings are conected by a semicarbazone fragment (C6/N2/N3/C7/O1/N4). The values of the dihedral angles between the aromatic rings and the semicarbazone fragnent are 23.99 (7)° and 42.15 (7)° for the benzene and pyridine rings, respectively. This indicates the lack of planarity.

The crystal packing is stabilized by a pair of strong intermolecular N—H···O hydrogen bonds conecting two molecules to form a centrosymmtric unit (*synthon*), and by an N—H···N hydrogen bond (Fig. 2), which extends the packing along the *c* axis (Fig 3). The crystal is also stabilized by intermolecular C—H···π interactions (Fig. 4). This type of interaction affects the conformation of the molecule, specifically the torsion angle between the benzene ring and the semicarbazone moeity. The molecular conformation is stabilized by intramolecular N4—H4N···N2 and C13—H13···O1 interactions (Table 1).

From the centroid–centroid distance between two pyridine rings [4.0085 (2)Å] and the angle between the normal of the aromatic plane and the centroid–centroid vector [35.13 (5)°], we conclude that there is no significant π-π stacking interaction between the pyridine rings.

### Experimental

A solution of 4-pyridine carboxaldehyde (1.0711 g, 0.01 mol) and 4-phenylsemicarbazide (1.5117 g, 0.01 mol) in absolute methanol (50 ml) was refluxed for 4 h in the presence of *p*-toluenesulfonic acid as catalyst, with continuous stirring. On cooling to room temperature the precipitate was filtered off, washed with copious cold methanol and dried in air (m.p. 493.15 K). White single crystals of compound (I) were obtained after recrystallization from a solution in methanol.

### Refinement

The NH and Schiff base CH H-atoms were found in difference Fourier maps and were freely refined: N3—H = 0.93 (2) Å, N4—H = 0.91 (2) Å and C6—H=0.99 (2) Å. All other C-bound H-atoms were included in calculated positions and treated as riding atoms: C—H = 0.93 Å for aromatic CH with U_iso_(H) = 1.2 × U_eq_(C). At the end of the refinement the highest peak in the electron density was 0.20 eÅ ^-3^, while the deepest hole was -0.17 eÅ ^-3^.

### Crystal data

**Table:**

C_13_H_12_N_4_O	*F*(000) = 504
*M**_r_* = 240.27	*D*_x_ = 1.320 Mg m^−^^3^
Monoclinic, *P*2_1_/*c*	Melting point: 493.15 K
Hall symbol: -P 2ybc	Cu *K*α radiation, λ = 1.54180 Å
*a* = 9.2794 (6) Å	Cell parameters from 2114 reflections
*b* = 10.3384 (8) Å	θ = 3.5–70.7°
*c* = 12.8244 (8) Å	µ = 0.72 mm^−^^1^
β = 100.744 (6)°	*T* = 295 K
*V* = 1208.73 (15) Å^3^	Rod, white
*Z* = 4	0.30 × 0.11 × 0.06 mm

### Data collection

**Table:**

Oxford Diffraction Xcalibur Gemini R diffractometer	2310 independent reflections
Radiation source: Enhance (Cu) X-ray Source	1702 reflections with *I* > 2σ(*I*)
graphite	*R*_int_ = 0.066
Detector resolution: 10.2673 pixels mm^-1^	θ_max_ = 70.8°, θ_min_ = 4.9°
ω scans	*h* = −10→11
Absorption correction: multi-scan (ABSPACK in *CrysAlis PRO*; Oxford Diffraction, 2010)	*k* = −12→11
*T*_min_ = 0.888, *T*_max_ = 1.000	*l* = −15→15
7257 measured reflections	

### Refinement

**Table:**

Refinement on *F*^2^	Secondary atom site location: difference Fourier map
Least-squares matrix: full	Hydrogen site location: inferred from neighbouring sites
*R*[*F*^2^ > 2σ(*F*^2^)] = 0.052	H atoms treated by a mixture of independent and constrained refinement
*wR*(*F*^2^) = 0.143	*w* = 1/[σ^2^(*F*_o_^2^) + (0.0648*P*)^2^ + 0.0647*P*] where *P* = (*F*_o_^2^ + 2*F*_c_^2^)/3
*S* = 1.06	(Δ/σ)_max_ < 0.001
2310 reflections	Δρ_max_ = 0.20 e Å^−^^3^
176 parameters	Δρ_min_ = −0.17 e Å^−^^3^
0 restraints	Extinction correction: *SHELXL*, Fc^*^=kFc[1+0.001xFc^2^λ^3^/sin(2θ)]^-1/4^
Primary atom site location: structure-invariant direct methods	Extinction coefficient: 0.0051 (8)

### Special details

**Table:**

Geometry. All esds (except the esd in the dihedral angle between two l.s. planes) are estimated using the full covariance matrix. The cell esds are taken into account individually in the estimation of esds in distances, angles and torsion angles; correlations between esds in cell parameters are only used when they are defined by crystal symmetry. An approximate (isotropic) treatment of cell esds is used for estimating esds involving l.s. planes.
Refinement. Refinement of F^2^ against ALL reflections. The weighted R-factor wR and goodness of fit S are based on F^2^, conventional R-factors R are based on F, with F set to zero for negative F^2^. The threshold expression of F^2^ > 2sigma(F^2^) is used only for calculating R-factors(gt) etc. and is not relevant to the choice of reflections for refinement. R-factors based on F^2^ are statistically about twice as large as those based on F, and R- factors based on ALL data will be even larger.

### Fractional atomic coordinates and isotropic or equivalent isotropic displacement parameters (Å^2^)

**Table:**

	*x*	*y*	*z*	*U*_iso_*/*U*_eq_	
O1	0.31018 (14)	0.04113 (16)	0.96710 (10)	0.0562 (4)	
N2	0.47959 (17)	−0.03751 (17)	0.75432 (12)	0.0474 (4)	
N3	0.45910 (18)	−0.01377 (19)	0.85540 (13)	0.0524 (5)	
N4	0.23363 (17)	0.07764 (18)	0.78923 (13)	0.0478 (4)	
N1	0.7010 (2)	−0.1407 (2)	0.43544 (14)	0.0619 (5)	
C7	0.3301 (2)	0.0358 (2)	0.87508 (14)	0.0443 (5)	
C4	0.5324 (2)	−0.1475 (2)	0.55575 (16)	0.0505 (5)	
H4	0.4383	−0.1666	0.5665	0.061*	
C3	0.5689 (2)	−0.1643 (2)	0.45723 (16)	0.0557 (5)	
H3	0.4964	−0.1940	0.4024	0.067*	
C8	0.0926 (2)	0.1288 (2)	0.79070 (15)	0.0467 (5)	
C5	0.6378 (2)	−0.1016 (2)	0.63847 (15)	0.0466 (5)	
C9	0.0279 (2)	0.2034 (2)	0.70528 (16)	0.0551 (5)	
H9	0.0782	0.2193	0.6502	0.066*	
C6	0.6074 (2)	−0.0739 (2)	0.74389 (16)	0.0489 (5)	
C13	0.0161 (2)	0.1054 (2)	0.87145 (16)	0.0575 (6)	
H13	0.0572	0.0541	0.9289	0.069*	
C10	−0.1102 (3)	0.2545 (3)	0.70086 (19)	0.0672 (7)	
H10	−0.1528	0.3038	0.6425	0.081*	
C2	0.8014 (2)	−0.0989 (3)	0.51620 (18)	0.0657 (6)	
H2	0.8949	−0.0817	0.5034	0.079*	
C1	0.7765 (2)	−0.0794 (2)	0.61736 (17)	0.0571 (6)	
H1	0.8519	−0.0516	0.6709	0.069*	
C12	−0.1218 (2)	0.1586 (3)	0.86654 (19)	0.0687 (7)	
H12	−0.1722	0.1434	0.9216	0.082*	
C11	−0.1858 (3)	0.2335 (3)	0.7820 (2)	0.0712 (7)	
H11	−0.2783	0.2692	0.7796	0.085*	
H6	0.686 (2)	−0.083 (2)	0.8069 (17)	0.058 (6)*	
H3N	0.533 (3)	−0.031 (2)	0.9132 (19)	0.065 (7)*	
H4N	0.266 (2)	0.080 (2)	0.7265 (18)	0.053 (6)*	

### Atomic displacement parameters (Å^2^)

**Table:**

	*U*^11^	*U*^22^	*U*^33^	*U*^12^	*U*^13^	*U*^23^
O1	0.0482 (8)	0.0824 (11)	0.0385 (7)	0.0050 (7)	0.0096 (6)	−0.0017 (7)
N2	0.0489 (9)	0.0571 (10)	0.0367 (8)	0.0029 (7)	0.0089 (7)	−0.0020 (7)
N3	0.0444 (9)	0.0765 (13)	0.0358 (8)	0.0107 (8)	0.0066 (7)	−0.0017 (8)
N4	0.0439 (9)	0.0619 (11)	0.0388 (8)	0.0066 (7)	0.0110 (7)	0.0011 (7)
N1	0.0681 (12)	0.0707 (13)	0.0507 (10)	0.0103 (9)	0.0212 (9)	0.0032 (9)
C7	0.0430 (10)	0.0525 (12)	0.0373 (10)	−0.0012 (8)	0.0076 (7)	−0.0035 (8)
C4	0.0517 (11)	0.0529 (12)	0.0482 (11)	0.0021 (9)	0.0124 (9)	−0.0005 (9)
C3	0.0614 (13)	0.0577 (14)	0.0473 (12)	0.0047 (10)	0.0081 (9)	−0.0026 (9)
C8	0.0447 (10)	0.0521 (12)	0.0437 (10)	0.0019 (8)	0.0092 (8)	−0.0038 (8)
C5	0.0484 (10)	0.0497 (12)	0.0423 (10)	0.0084 (8)	0.0103 (8)	0.0044 (8)
C9	0.0580 (12)	0.0613 (14)	0.0472 (11)	0.0101 (10)	0.0127 (9)	0.0036 (10)
C6	0.0430 (10)	0.0603 (13)	0.0428 (10)	0.0061 (9)	0.0065 (8)	0.0024 (9)
C13	0.0521 (11)	0.0751 (16)	0.0479 (11)	0.0077 (10)	0.0159 (9)	0.0106 (10)
C10	0.0679 (14)	0.0759 (17)	0.0579 (14)	0.0233 (11)	0.0116 (11)	0.0102 (11)
C2	0.0568 (13)	0.0857 (18)	0.0597 (13)	0.0021 (11)	0.0239 (11)	0.0007 (12)
C1	0.0464 (11)	0.0726 (15)	0.0530 (12)	0.0040 (10)	0.0105 (9)	−0.0012 (10)
C12	0.0568 (13)	0.0924 (19)	0.0611 (14)	0.0139 (12)	0.0219 (10)	0.0058 (13)
C11	0.0586 (14)	0.0851 (19)	0.0725 (16)	0.0239 (12)	0.0193 (12)	0.0045 (13)

### Geometric parameters (Å, °)

**Table:**

O1—C7	1.229 (2)	C8—C9	1.382 (3)
N2—C6	1.275 (2)	C5—C1	1.383 (3)
N2—N3	1.367 (2)	C5—C6	1.460 (3)
N3—N2	1.367 (2)	C9—C10	1.378 (3)
N3—C7	1.368 (2)	C9—H9	0.9300
N3—H3N	0.93 (2)	C6—H6	0.99 (2)
N4—C7	1.354 (2)	C13—C12	1.384 (3)
N4—C8	1.415 (2)	C13—H13	0.9300
N4—H4N	0.91 (2)	C10—C11	1.376 (3)
N1—C3	1.329 (3)	C10—H10	0.9300
N1—C2	1.329 (3)	C2—C1	1.374 (3)
C4—C3	1.379 (3)	C2—H2	0.9300
C4—C5	1.386 (3)	C1—H1	0.9300
C4—H4	0.9300	C12—C11	1.373 (3)
C3—H3	0.9300	C12—H12	0.9300
C8—C13	1.381 (3)	C11—H11	0.9300
			
C6—N2—N3	116.68 (16)	C10—C9—H9	119.7
N2—N3—C7	121.56 (16)	C8—C9—H9	119.7
N2—N3—H3N	120.5 (14)	N2—C6—C5	119.94 (17)
C7—N3—H3N	117.9 (14)	N2—C6—H6	120.3 (12)
C7—N4—C8	125.56 (16)	C5—C6—H6	119.8 (12)
C7—N4—H4N	116.7 (13)	C8—C13—C12	119.8 (2)
C8—N4—H4N	117.4 (13)	C8—C13—H13	120.1
C3—N1—C2	115.80 (18)	C12—C13—H13	120.1
O1—C7—N4	124.85 (17)	C11—C10—C9	120.6 (2)
O1—C7—N3	119.15 (17)	C11—C10—H10	119.7
N4—C7—N3	115.99 (16)	C9—C10—H10	119.7
C3—C4—C5	119.03 (19)	N1—C2—C1	124.5 (2)
C3—C4—H4	120.5	N1—C2—H2	117.7
N1—C3—C4	124.3 (2)	C1—C2—H2	117.7
N1—C3—H3	117.9	C2—C1—C5	119.1 (2)
C4—C3—H3	117.9	C2—C1—H1	120.4
C13—C8—C9	118.96 (18)	C5—C1—H1	120.4
C13—C8—N4	123.37 (18)	C11—C12—C13	121.3 (2)
C9—C8—N4	117.65 (18)	C11—C12—H12	119.3
C1—C5—C4	117.19 (18)	C13—C12—H12	119.3
C1—C5—C6	119.74 (18)	C12—C11—C10	118.7 (2)
C4—C5—C6	123.05 (18)	C12—C11—H11	120.7
C10—C9—C8	120.6 (2)	C10—C11—H11	120.7
			
C6—N2—N3—C7	174.5 (2)	N3—N2—C6—C5	179.75 (18)
C8—N4—C7—O1	−2.8 (3)	C1—C5—C6—N2	148.6 (2)
C8—N4—C7—N3	178.13 (19)	C4—C5—C6—N2	−30.0 (3)
N2—N3—C7—O1	171.39 (19)	C9—C8—C13—C12	−1.2 (3)
N2—N3—C7—N4	−9.5 (3)	N4—C8—C13—C12	−179.6 (2)
C2—N1—C3—C4	0.3 (3)	C8—C9—C10—C11	0.7 (4)
C5—C4—C3—N1	0.8 (3)	C3—N1—C2—C1	−0.1 (4)
C7—N4—C8—C13	−21.7 (3)	N1—C2—C1—C5	−1.3 (4)
C7—N4—C8—C9	159.8 (2)	C4—C5—C1—C2	2.3 (3)
C3—C4—C5—C1	−2.1 (3)	C6—C5—C1—C2	−176.3 (2)
C3—C4—C5—C6	176.49 (19)	C8—C13—C12—C11	0.8 (4)
C13—C8—C9—C10	0.4 (3)	C13—C12—C11—C10	0.3 (4)
N4—C8—C9—C10	179.0 (2)	C9—C10—C11—C12	−1.1 (4)

### Hydrogen-bond geometry (Å, °)

**Table:**

Cg2 is the centroid of the C8–C13 ring.

**Table:**

*D*—H···*A*	*D*—H	H···*A*	*D*···*A*	*D*—H···*A*
N3—H3N···O1^i^	0.93 (2)	1.91 (2)	2.833 (2)	172 (2)
N4—H4N···N1^ii^	0.91 (2)	2.24 (2)	3.122 (3)	161.8 (19)
N4—H4N···N2	0.91 (2)	2.29 (2)	2.685 (2)	105.4 (16)
C13—H13···O1	0.93	2.31	2.854 (2)	117
C1—H1···Cg2^iii^	0.93	2.88	3.644 (2)	140

Symmetry codes: (i) −*x*+1, −*y*, −*z*+2; (ii) −*x*+1, −*y*, −*z*+1;  (iii) *x*+1, *y*, *z*.
